# Hierarchical assembly of the MLL1 core complex regulates H3K4 methylation and is dependent on temperature and component concentration

**DOI:** 10.1016/j.jbc.2023.102874

**Published:** 2023-01-06

**Authors:** Kevin E.W. Namitz, Song Tan, Michael S. Cosgrove

**Affiliations:** 1 State University of New York (SUNY) Upstate Medical University, Department of Biochemistry and Molecular Biology, Syracuse, NY, USA; 2 Penn State University, Department of Biochemistry and Molecular Biology, University Park, PA, USA

**Keywords:** analytical ultracentrifugation, histone methylation, thermodynamics, epigenetics, enzyme kinetics, H3K4, histone H3 lysine 4, H3K4me1, H3K4 monomethylation, MLL, mixed lineage leukemia, SEC, size-exclusion chromatography, SET, SuVar, Ez, Trx, SV-AUC, sedimentation velocity analytical ultracentrifugation, WRAD_2_, WDR5, RbBP5, Ash2L, and two copies of DPY-30

## Abstract

Enzymes of the mixed lineage leukemia (MLL) family of histone H3 lysine 4 (H3K4) methyltransferases are critical for cellular differentiation and development and are regulated by interaction with a conserved subcomplex consisting of WDR5, RbBP5, Ash2L, and DPY30. While pairwise interactions between complex subunits have been determined, the mechanisms regulating holocomplex assembly are unknown. In this investigation, we systematically characterized the biophysical properties of a reconstituted human MLL1 core complex and found that the MLL1–WDR5 heterodimer interacts with the RbBP5–Ash2L–DPY30 subcomplex in a hierarchical assembly pathway that is highly dependent on concentration and temperature. Surprisingly, we found that the disassembled state is favored at physiological temperature, where the enzyme rapidly becomes irreversibly inactivated, likely because of complex components becoming trapped in nonproductive conformations. Increased protein concentration partially overcomes this thermodynamic barrier for complex assembly, suggesting a potential regulatory mechanism for spatiotemporal control of H3K4 methylation. Together, these results are consistent with the hypothesis that regulated assembly of the MLL1 core complex underlies an important mechanism for establishing different H3K4 methylation states in mammalian genomes.

Cellular identity in multicellular organisms is maintained in part by enzymes that regulate the degree of histone H3 lysine 4 (H3K4) methylation (1). Dimethylation and trimethylation of H3K4 (H3K4me2,3) are enriched in gene bodies and promoters of active genes (2, 3, 4), respectively, and function to recruit nucleosome-remodeling complexes that regulate transcription (5, 6, 7, 8, 9). H3K4 monomethylation (H3K4me1) is associated with not only active gene enhancers (10, 11, 12) but also with gene silencing (13, 14, 15, 16, 17). Because genome-wide alterations in the patterns of H3K4 methylation are linked to the aberrant transcriptional programs in developmental disorders and cancers (18, 19, 20, 21, 22, 23, 24, 25, 26, 27, 28), there is significant interest in understanding how different H3K4 methylation states are established and maintained.

Mixed lineage leukemia protein-1 (MLL1, ALL1, HRX, and KMT2C) is a member of the SET1 family of H3K4 methyltransferases and is frequently altered in poor prognosis acute leukemias (29). MLL1 is a large protein with 3969 amino acids and assembles into a supercomplex with ∼30 subunits (30, 31, 32, 33). Subunits shared among all SET1 family members include WDR5, RbBP5, Ash2L, and two copies of DPY-30 (WRAD_2_), which associate into a subcomplex that interacts with the C-terminal SuVar, Ez, Trx (SET) domain of MLL1 (34, 35, 36, 37, 38). *In vitro* studies have shown that the MLL1 SET domain predominantly catalyzes H3K4 monomethylation (36), whereas multiple methylation depends on interaction of MLL1 with WRAD_2_, forming what is known as the MLL1 core complex (also known as human COMPASS or MWRAD_2_) (34, 36, 39). The requirement of full MWRAD_2_ complex for optimal enzymatic activity suggests that H3K4 methylation may be regulated at the level of subunit assembly in the cell. Consistent with this hypothesis, genome-wide studies show that, while MLL1 localizes to thousands of genes in mammalian genomes, multiple methylation of H3K4 is mainly correlated with the subset of genes where MLL1 colocalizes with WRAD_2_ subunits (40). In addition, disease-specific missense mutations have been shown to disrupt MLL family core complexes (41), suggesting that aberrations in complex assembly may be associated with human disease. More recently, several laboratories have shown that perturbation of MLL1 core complex assembly with protein–protein interaction inhibitors may have utility as a novel therapeutic approach for treating malignancies (42, 43, 44). Together, these results suggest that knowledge of the molecular mechanisms controlling MLL1 core complex assembly will be crucial for understanding of how different H3K4 methylation states are regulated in mammalian genomes. However, progress has been impeded by the lack of understanding of the biophysical and thermodynamic mechanisms involved.

Biochemical reconstitution studies using a minimal MLL1 SET domain construct show that the stoichiometry of the MLL1 core complex consists of one copy of the MLL1, WDR5, RbBP5, and Ash2L subunits, and two copies of the DPY-30 subunit (MWRAD_2_) forming a complex with a mass of ∼205 kDa (36). Direct interactions have been observed between MLL1 and WDR5 (35, 37, 45), WDR5 and RbBP5 (46, 47), RbBP5 and Ash2L (36), and Ash2L and DPY30 (36, 48, 49). While these pairwise interactions suggest a linear arrangement of subunits, several lines of evidence indicate a more intricate quaternary structure. For example, while MLL1 does not interact with RbBP5 or Ash2L in pairwise experiments (36), an investigation of Kabuki syndrome missense mutations suggests that the MLL1 SET domain directly interacts with the RbBP5–Ash2L heterodimer within the context of the holocomplex (41). The WDR5 subunit functions to stabilize this interaction by directly binding to the MLL1 WDR5 interaction motif (35, 37, 45) and RbBP5 (34, 36). Binding experiments show that the weakest pairwise interaction occurs between the WDR5 and RbBP5 subunits (36), suggesting the complex may be hierarchically assembled. These interactions have been confirmed in recent cryo-EM and X-ray crystal structures of related SET1 family complexes (50, 51, 52, 53). Together, these results suggest that complex assembly is hierarchical in nature, with the requirement for the formation of distinct subcomplexes before assembly of the higher-order quaternary structure. The choreographic details of this assembly pathway are unknown.

In this investigation, to better understand the MLL1 core complex assembly pathway, we systematically characterized the hydrodynamic and kinetic properties of a reconstituted human MLL1 core complex under a variety of conditions. We found that MLL1 core complex assembly is highly concentration and temperature dependent. Consistent with the hypothesized hierarchical assembly pathway, we found that the holocomplex assembles through interactions between the MW and RAD_2_ subcomplexes, and that MWRAD_2_ formation is correlated with enzymatic activity. Surprisingly, we found that the disassembled state is favored at physiological temperatures and at concentrations typically used in steady-state enzymatic assays. This result suggests that the complex is predominantly disassembled in a cellular context and is then assembled in regions with high local concentrations of complex components. This is consistent with the hypothesis that regulated assembly of the MLL1 core complex underlies an important mechanism for establishing different H3K4 methylation states in mammalian genomes. It also suggests that regulated assembly of chromatin-modifying and/or chromatin-remodeling complexes may be an additional layer of control over these important cellular processes, further fine-tuning their actions within the nucleus.

## Results

### MLL1 core complex assembly is concentration and temperature dependent

**Figure 1:**
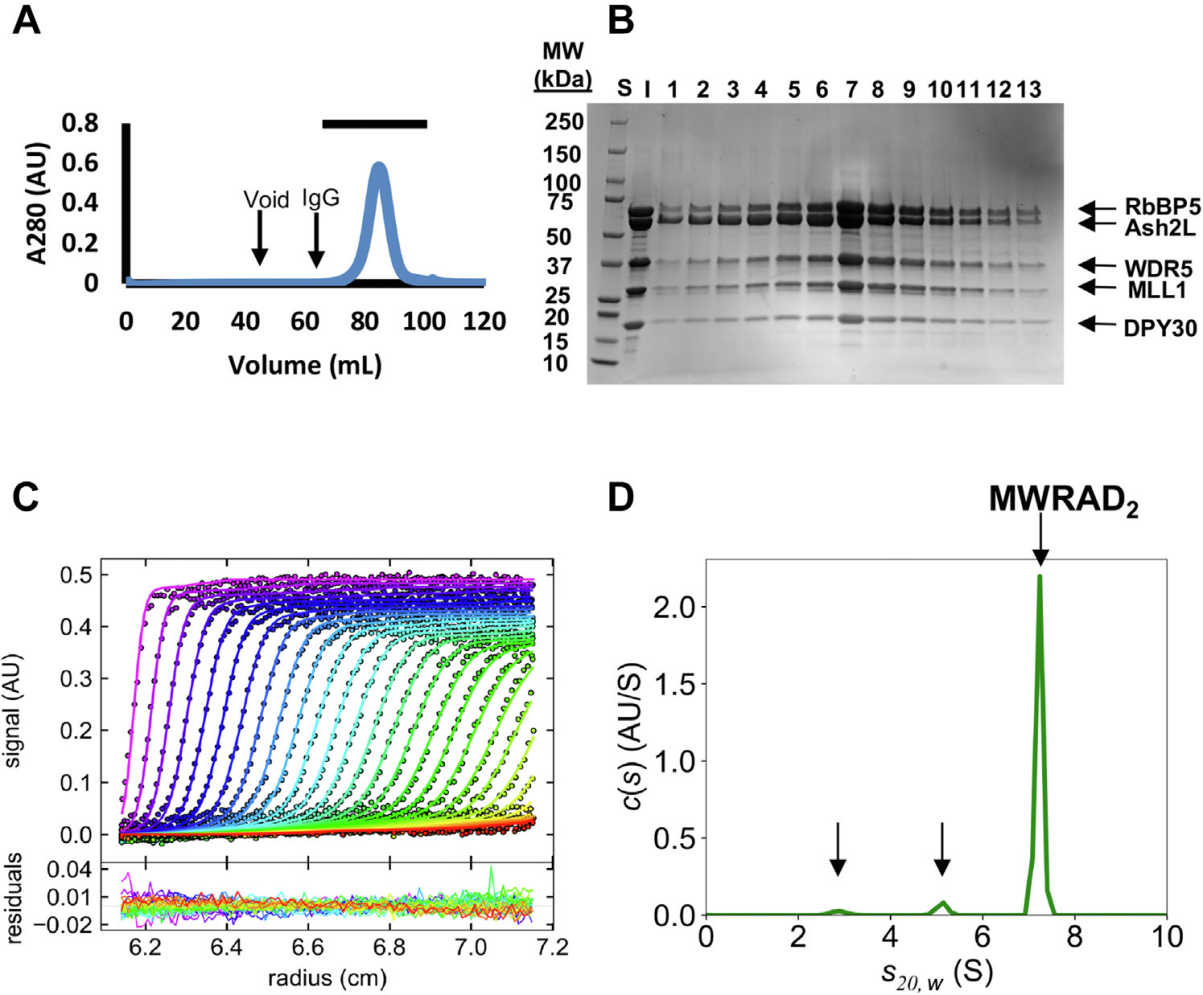
**Biophysical characterization of the MLL1 core complex.***A,* chromatogram of S200 SEC-purified MWRAD_2_. The void volume and elution volume of IgG (M_r_ = 158 kDa) are indicated. The *horizontal bar* above the peak indicates fractions shown on the Coomassie-stained SDS-PAGE gel in (*B*). Also in *B*, “S” stands for standard and “I” stands for input control. *C, upper panel,* SV-AUC run showing raw data (points) and fits using the continuous distribution (*c*(*s*)) method by the program SEDFIT (*solid lines*) (56). Each line from *left* to *right* shows the absorbance profile of distinct time points taken throughout the experiment. The *lower panel* shows the residuals derived from the fit. Shown is a typical run of 5 μM MWRAD_2_ taken at 5 °C. *D,* diffusion-deconvolved sedimentation coefficient distribution (*c*(*s*)) obtained using the fits to the raw data shown in (*C*). All profiles are shown with experimental *s∗* values corrected to standard conditions at 20 °C in water (*s*_*20,w*_ (*S*)). The sedimentation positions of MWRAD_2_ and the two minor peaks are indicated with *arrows*. IgG, immunoglobulin G; MLL, mixed lineage leukemia; MWRAD_2_, MLL core complex with WDR5, RbBP5, Ash2L, and two copies of DPY-30; SEC, size-exclusion chromatography; SV-AUC, sedimentation velocity analytical ultracentrifugation.

**Figure 2:**
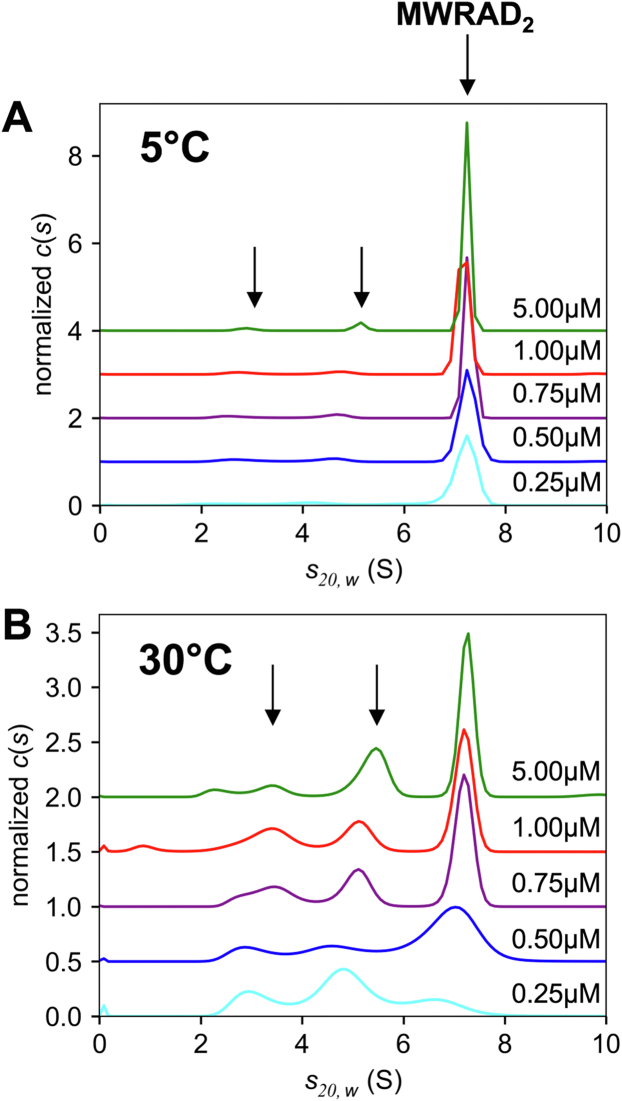
**The holo–MLL1 core complex assembles from two subcomplexes.***A, c*(*s*) distributions of MWRAD_2_ at 5 °C at five different concentrations: 0.25 μM (*cyan*), 0.5 μM (*blue*), 0.75 μM (*purple*), 1.0 μM (*red*), and 5.0 μM (*green*). Each profile was normalized by total integrated area under the peaks. *B,* the same as in (*A*) but at 30 °C. The *unlabeled arrows* in both (*A* and *B*) indicate the positions of the putative subcomplex peaks at the highest concentration. MLL, mixed lineage leukemia; MWRAD_2_, MLL core complex with WDR5, RbBP5, Ash2L, and two copies of DPY-30.

To better understand MLL1 core complex assembly, we purified human recombinant MWRAD_2_ as described in the Experimental procedures section and characterized its oligomeric behavior by size-exclusion chromatography (SEC) and sedimentation velocity analytical ultracentrifugation (SV-AUC). SEC revealed that the purified complex eluted as a single symmetrical peak (Fig. 1*A*), and SDS-PAGE of the indicated fractions showed the presence of all five subunits with the expected stoichiometry (Fig. 1*B*). We note that the complex elutes later than expected based on its theoretical mass, which is likely because of the significant shape asymmetry of the particle. We then chose SV-AUC to characterize the concentration and temperature dependence of the complex in solution. SV-AUC is a first-principle technique that measures the time course of sedimentation of macromolecules in an artificial gravitational field in a way that maintains the equilibrium of reversible associations—allowing extraction of equilibrium and kinetic properties of interactions (54, 55). Sedimentation boundaries formed as the particles sediment over time were fit using a finite element analysis of Lamm equation solutions (Fig. 1*C*) (56) to give the diffusion-deconvoluted sedimentation coefficient distribution *c*(*s*) (Fig. 1*D*). The *c*(*s*) plot of MWRAD_2_ at 5 μM loading concentration at 5 °C revealed a large peak accounting for almost 90% of the signal with an *s*_*20,w*_ (*S*) value of 7.2 and two minor peaks at 2.9 and 4.7 *S* that each account for 4 to 5% of the signal (noted with *arrows* in Fig. 1*D*). The major peak at 7.2 *S* corresponds to the fully assembled MLL1 core complex, which we previously showed assembles with a stoichiometry of 1:1:1:1:2 for the MWRAD_2_ subunits, respectively (36). In addition, the *S* value of MWRAD_2_ is independent of loading concentration (Fig. 2*A*), indicating that the complex is stable at 5 °C and has a relatively long lifetime compared with the timescale of sedimentation (57). Using the derived weight-averaged frictional coefficient (*f/f*_*0*_) of 1.7, the calculated molecular mass from this *S* value was 209,561 Da, which is within error of the expected mass (205,402) based on the amino acid sequence of the holocomplex subunits at the indicated stoichiometry.

The minor peaks observed in the *c*(*s*) distribution in Figure 1*D* could represent trace contaminants in the sample or minor populations of dissociated subcomplexes and/or subunits. To distinguish these hypotheses, we compared *c*(*s*) distributions of MWRAD_2_ at concentrations ranging from 0.25 to 5 μM at 5 °C (Fig. 2*A*) and 30 °C (Fig. 2*B*). If the minor peaks represent noninteracting contaminants, then the relative amount of signal between the major and minor peaks will not vary as the loading concentration is decreased. In contrast, if the complex is dissociating into subcomplexes, then the relative amount of signal in the major and minor peaks will change as the loading concentration is varied. The results were consistent with the latter possibility. For example, while the effect at 5 °C was modest, when the loading concentration of the complex was decreased from 5 to 0.25 μM, the amount of signal corresponding to the holocomplex decreased from ∼88% to ∼83% of the total signal, with a corresponding increase in both minor peak signals (Fig. 2*A*). The effect was more obvious at 30 °C, which showed that the signal corresponding to the holocomplex decreased from 65% to 25% of the total signal as the loading concentration was decreased (Fig. 2*B*). These results suggest that the minor peaks represent dissociated subcomplexes and/or subunits. Furthermore, because the *S* values of the minor peaks show varying degrees of concentration dependence, they likely represent subcomplex reaction boundaries as opposed to individual noninteracting subunits. These data suggest that the holocomplex assembles from predominantly two subcomplexes in a temperature- and concentration-dependent manner.

### The MLL1 core complex assembles from MW and RAD_2_ subcomplexes

Previous experiments suggested that the holocomplex is assembled by pairwise interactions as follows: M ⇌ W ⇌ R ⇌ A ⇌ D_2_ (36). Since the weakest pairwise interaction occurs between WDR5 and RbBP5 (36), we predicted that the complex assembles by first forming MW and RAD_2_ subcomplexes, which then interact to form the holocomplex (Scheme 1). However, we reasoned that there are at least two additional reaction schemes that could give rise to the three boundaries observed in the holocomplex *c*(*s*) profiles (Schemes 2 and 3). To distinguish among these schemes, a Bayesian approach was used to analyze the SV-AUC data of the holocomplex collected at 25 °C. The Bayesian approach is a variant of the standard maximum entropy regularization method utilized in the *c*(*s*) analysis in that, instead of assuming a uniform probability for the occurrence of species at every *S* value in a distribution, it utilizes prior information to assign different probabilities in different regions of *S* values (58). A key feature of the Bayesian implementation in SEDFIT (National Institutes of Health) is that it maintains the same degrees of freedom used in the standard *c*(*s*) analysis, and therefore, imperfections in the expected values will result in additional features in the *c*^(*p*)^(*s*) plots in order to maintain the quality of the fit (58). The Bayesian analysis therefore allows us to visually determine which reaction scheme gives a *c*^(p)^(*s*) profile that best fits the experimental data.

Scheme1: $M+W⇌MW,R+AD_{2}⇌RAD_{2},MW+RAD_{2}⇌MWRAD_{2}$

Scheme2: $M+W\;⇌MW+R\;⇌MWR+AD_{2}\;⇌MWRAD_{2}$

Scheme3: $W+R⇌WR+AD_{2}⇌WRAD_{2}+M\;⇌MWRAD_{2}$

**Figure 3:**
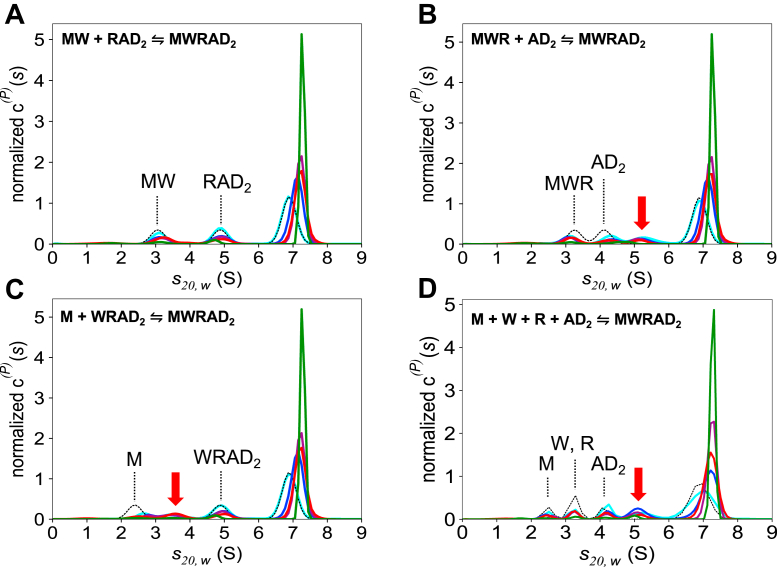
**The holo–MLL1 core complex assembles from MW and RAD**_**2**_**subcomplexes.** Bayesian analysis of MWRAD_2_ SV-AUC data collected at 25 °C. Maximum entropy regularizations were restrained with expected values (indicated with the *dotted line*) for each indicated subcomplex derived from separate experiments (Fig. S1 and Table S1) to give *c*^(*P*)^(*s*) distributions (*colored lines*), which were normalized by total integrated area. Concentrations of MWRAD_2_ in each run were 0.25 μM (*cyan*), 0.5 μM (*blue*), 0.75 μM (*purple*), 1.0 μM (*red*), and 5.0 μM (*green*). The *c*^(*P*)^(*s*) distributions used the following *S* values as prior expectations: (*A*) MW (3.0 *S*), RAD_2_ (4.4 *S*), and MWRAD_2_ (6.9 *S*) (Scheme 1); (*B*) MWR (3.3 *S*), AD_2_ (4.1 *S*), and MWRAD_2_ (6.9 *S*) (Scheme 2); (*C*) M (2.3 *S*), WRAD_2_ (4.4 *S*), and MWRAD_2_ (6.9 *S*) (Scheme 3); (*D*) M (2.3 *S*), W (3.2 *S*), R (3.4 *S*), AD_2_ (4.1 *S*), and MWRAD_2_ (6.9 *S*) (concerted assembly scheme). MLL, mixed lineage leukemia; MLL core complex with WDR5, RbBP5, Ash2L, and two copies of DPY-30; SV-AUC, sedimentation velocity analytical ultracentrifugation.

To obtain the expected *S* values for each of the predicted subcomplexes or subunits in each reaction scheme, we mixed stoichiometric amounts of their respective subunits and characterized their concentration dependence by SV-AUC at 25 °C (Fig. S1 and Table S1). We then used each of the *S* values collected at 0.25 μM as prior expectations in the Bayesian analysis of the holocomplex. As shown in Figure 3*A*, when the independently determined *S* values for MW, RAD_2_, and the MWRAD_2_ species were used as prior expectations in the Bayesian analysis of the holocomplex at 0.25 μM (*black dotted line*), three peaks in the *c*^(*p*)^(*s*) plot were observed that were in excellent agreement with the expectations (*cyan line*). Indeed, good agreement was observed using the same *S* values as prior expectations for Bayesian fits of the experimental data collected at higher holocomplex concentrations (Fig. 3*A*). The only deviation observed was for the position and amplitude of the holocomplex peak, which at 25 °C shifts from 6.8 to 7.2 *S* in a concentration-dependent manner (Fig. 3*A*). In contrast, when a similar analysis was conducted instead using the expected *S* values for the MWR and AD_2_ subcomplexes predicted by Scheme 2, additional features in the *c*^(*p*)^(*s*) plot with an *S* value of ∼5.3 were observed at all loading concentrations that did not match the prior expectations (Fig. 3*B*, *red arrow*). Similarly, using the expected *S* values for M and WRAD_2_ as predicted by Scheme 3, the *c*^(*p*)^(*s*) plot showed little evidence of a species matching the expected value of free MLL1 at 2.3 *S* and also showed additional features at ∼3.5 *S* that did not match expectations (Fig. 3*C*, *red arrow*). To test whether the holocomplex assembles in a concerted fashion from individual subunits, we also performed a similar Bayesian analysis using the predetermined *S* values for M, W, R, AD_2_, and MWRAD_2_ as prior expectations (AD_2_ is treated as a discrete species since it does not appreciably dissociate under the range of concentrations that can be detected by the absorbance optical system used in these experiments (36)). The *c*^(p*)*^(*s*) plot showed additional features with an *S* value of ∼5.2 that did not match expectations (Fig. 3*D*, *red arrow*). Together, these results are consistent with the hypothesis that MLL1 core complex is hierarchically assembled by association of MW and RAD_2_ subcomplexes.

### The disassembled state of the MLL1 core complex is favored at physiological temperature

**Figure 4:**
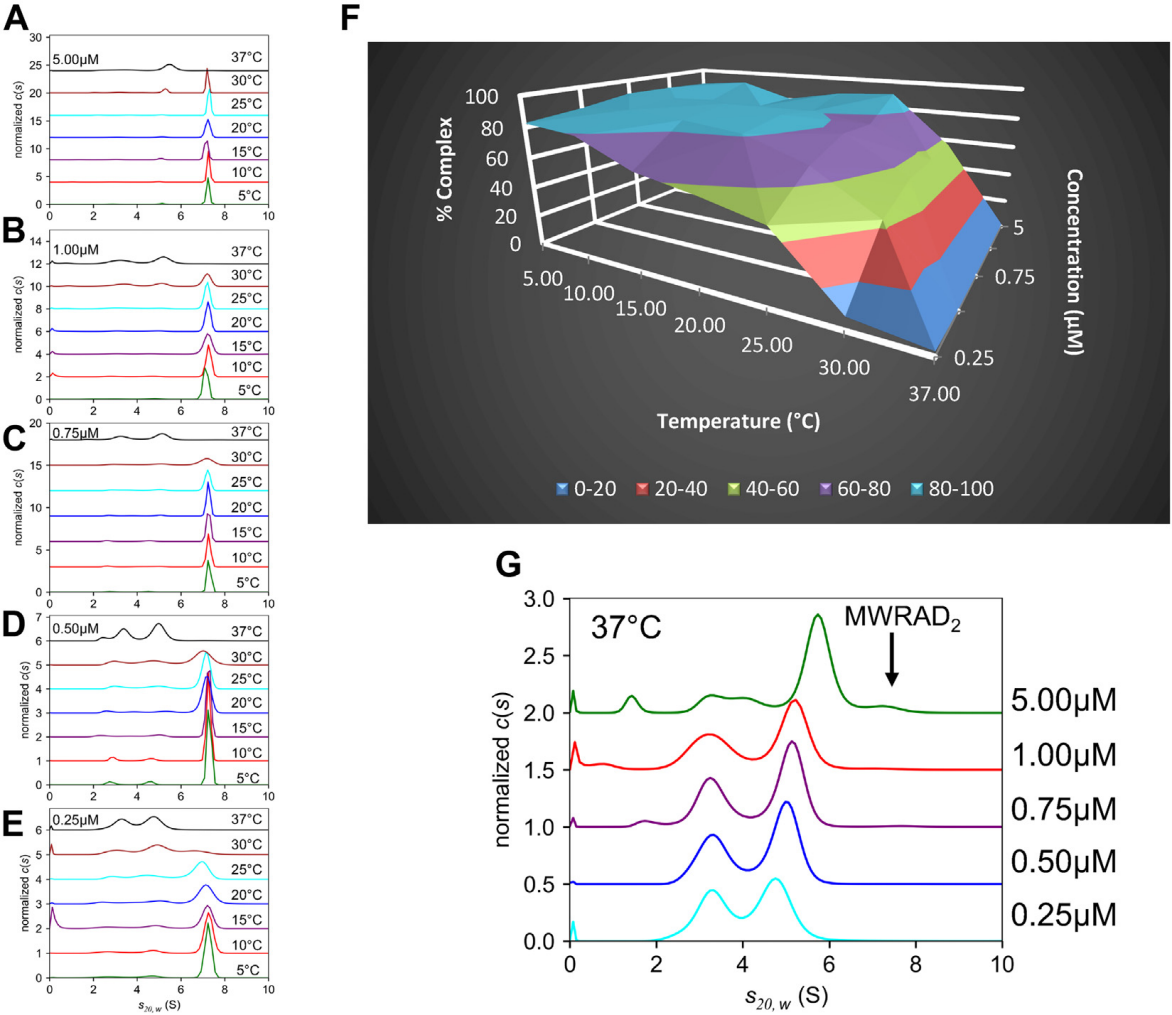
**Temperature dependence of MLL1 core complex assembly.***A*–*E,* representative *c*(*s*) distributions of the MLL1 core complex at the indicated temperatures and loading concentrations. Each distribution was normalized for total integrated area. *F,* surface plot summarizing the percentage of signal in SV-AUC experiments corresponding to the *S* value of the MLL1 core complex as a function of temperature and concentration (Table S2). These values were obtained as described in the *Experimental procedures* section. *G, c*(*s*) distributions from five MWRAD_2_ concentrations at 37 °C normalized by total integrated area (note: each distribution corresponds to the *black line* from the respective concentration panel in *A*–*E*). The position of holo-MWRAD_2_ at 7.2 *S* is indicated with the *arrow*. MLL, mixed lineage leukemia; MWRAD_2,_ MLL core complex with WDR5, RbBP5, Ash2L, and two copies of DPY-30; SV-AUC, sedimentation velocity analytical ultracentrifugation.

To further explore the thermodynamics of MLL1 core complex assembly, we compared the temperature dependence of MWRAD_2_ formation at several different loading concentrations using SV-AUC (Fig. 4). Each *c*(*s*) profile was integrated, and the relative amount of signal corresponding to the *S* value of the holocomplex was plotted as a function of temperature and total loading concentration (Fig. 4*F*). At the highest loading concentration (5 μM), little variation in the amount of holocomplex was observed between 5 and 25 °C (Fig. 4, *A* and *F*), with a peak that accounted for 81 to 92% of the total signal (Table S2). In contrast, at temperatures greater than 25 °C, the amount of holocomplex decreased precipitously until only ∼3% of the signal could be observed at 37 °C (Fig. 4, *A* and *F* and Table S2). The effect of temperature on MLL1 core complex stability became increasingly more severe as the loading concentration was decreased. For example, at the lowest loading concentration (0.25 μM), only the 5 and 10 °C runs showed ∼80% holocomplex (Fig. 4, *E* and *F* and Table S2); whereas at higher temperatures, the signal corresponding to the holocomplex decreased from ∼63% at 15 °C to ∼2% of the total signal at 37 °C (Fig. 4*F* and Table S2). At 37 °C, most of the signal is instead dominated by the two subcomplex peaks with *S* values of ∼3 and 4.7 (Fig. 4*G*). These data are consistent with the hypothesis that the holo–MLL1 core complex assembles from interaction of MW and RAD_2_, the equilibrium of which is highly concentration and temperature dependent.

Surprisingly, at all the concentrations tested, very little holocomplex with an *S* value of 7.2 was observed at physiological temperature (37 °C) (Fig. 4*G*). This suggests that the disassembled state of the MLL1 core complex may predominate in cells and that other factors are required to stabilize the assembled state. In support of this hypothesis, closer examination of the *c*(*s*) profiles of the complex at 37 °C revealed evidence that increased protein concentration promotes complex formation. For example, while similar amounts of signal are observed in the two subcomplex peaks at the 0.25 μM loading concentration (*cyan line*, Fig. 4*G*), the relative amount of signal in the two peaks changes with progressively higher concentrations. The intensity of the larger peak increased at the expense of the smaller peak and began to show evidence of concentration-dependent shifting to higher *S* values. This hydrodynamic behavior is consistent with a reaction boundary composed of free and bound reactants that interconvert under a rapid kinetic regime that cannot be resolved within the signal to noise of the experiment (57). These results suggest that, unlike the long lifetime of the assembled complex observed at 5 °C, the kinetics of the interaction have changed at 37 °C such that the complex now has a short lifetime compared with the timescale of sedimentation.

**Figure 5:**
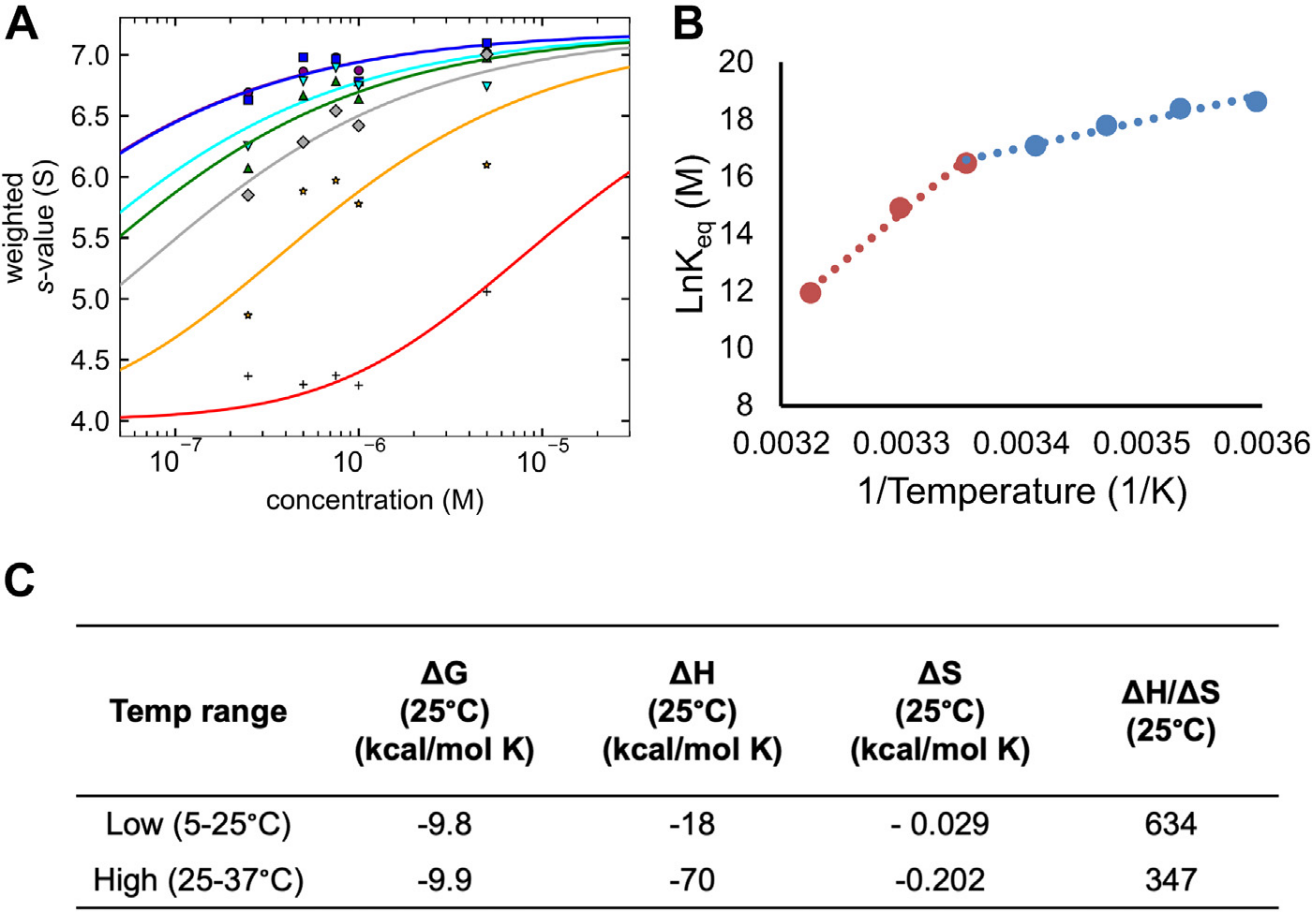
**Thermodynamic characterization of MLL1 core complex assembly.***A,* signal-weighted (*s*_*w*_) isotherms of MWRAD_2_ were obtained for each temperature, plotted against loading concentration, and fit to an A + B ⇋ AB heteroassociation model using SEDPHAT. The *lines* represent the fits for each isotherm, which were conducted at 5 °C (*blue*), 10 °C (*purple*), 15 °C (*cyan*), 20 °C (*green*), 25 °C (*gray*), 30 °C (*orange*), and 37 °C (*red*). *K*_*d*_^*app*^ values are summarized in Table 1. *B,* van’t Hoff plot derived from the apparent *K*_*eq*_ values. Linear regression was used to independently fit the data for the high temperature range (*red*, 25–37 °C) and low temperature range (*blue*, 5–25 °C). *C,* summary of thermodynamic parameters for MLL1 core complex assembly under high and low temperature regimes derived from the van’t Hoff analysis in (*B*). MLL, mixed lineage leukemia; MWRAD_2,_ MWRAD_2_, MLL core complex with WDR5, RbBP5, Ash2L, and two copies of DPY-30.

We next analyzed the concentration series at each temperature to derive binding isotherms. We integrated each *c*(*s*) profile (between 0.5 and 9.5 *S*) to determine the weight-average sedimentation coefficients (*s*_*w*_) (59), which were then plotted against MWRAD_2_ concentration and fit to derive the apparent dissociation constant (*K*_*d*_^*app*^) for each isotherm (Fig. 5*A*). Given that the majority of signal in each *c*(*s*) profile could be assigned to three peaks, we applied the A + B ⇆ AB heteroassociation model in the program SEDPHAT (National Institutes of Health) (60) and obtained reasonable fits (Table 1). The derived *K*_*d*_^*app*^ values ranged from 7 nM at 5 °C to ∼6200 nM at 37 °C (Table 1). A van’t Hoff analysis showed that complex formation is exothermic, which is offset by the negative entropy change as the complex subunits become more ordered (Fig. 5, *B* and *C*). However, the van’t Hoff plot reveals a nonlinear relationship between *K*_eq_ and temperature, indicating a change in the heat capacity of the system at higher temperatures (Fig. 5*B*). These data suggest at least two mechanisms for complex assembly, which differ by temperature. At low temperatures (≤25 °C), the equilibrium favors complex formation, with a relatively long lifetime that is stable on the timescale of sedimentation. Under this mechanism, the interaction is dominated by enthalpic contributions to the free energy (Fig. 5*C*). At high temperatures (>25 °C), the equilibrium is shifted into the rapid kinetic regime with a short complex lifetime where dissociation is more likely. While there is little difference in the Gibbs free energy between mechanisms, there is a difference in the contributions between the enthalpic and entropic terms. At higher temperatures, the entropic penalty to complex formation was increased sevenfold compared with that of the lower temperature mechanism, whereas the difference in the enthalpic contribution was only increased by 3.8-fold (Fig. 5*C*). These results suggest that, at physiological temperature, one or more of the subunits samples alternate conformational states, some of which are not competent for complex assembly. However, given the observation that some holocomplex forms in a concentration-dependent manner, increased local concentration of subunits may be a mechanism that cells use to overcome the increased entropic cost of complex formation at 37 °C.

**Table 1:** Summary of apparent dissociation constants for MLL1 core complex assembly at different temperatures^a^

Temperature (°C)	*K*_*d*_^*app*^ (nM)	Confidence interval (1σ)
5	7	6–9
10	7	5–11
15	20	13–30
20	30	22–39
25	62	51–72
30	290	204–417
37	6200	4900–7900

a Dissociation constants and error estimates were obtained from fitting MWRAD_2_ concentration *versus* signal weight-average sedimentation coefficient (*s*_*w*_) using the A + B ⇋ AB heteroassociation model in SEDPHAT (59).

### Enzymatic activity of the MLL1 core complex is directly related to complex assembly

To determine the impact of concentration and temperature on the enzymatic activity of the MLL1 core complex, we incubated MWRAD_2_ (0.25–5 μM) with a fixed concentration of histone H3 peptide (10 μM) and saturating amounts of AdoMet (250 μM) at various temperatures. We then measured methylation using a label-free quantitative MALDI-TOF mass spectrometry assay (36). MALDI spectra were integrated, and the relative amount of each peptide species was plotted as a function of time. Data were fit using a numerical integration of rate equations approach implemented in KinTek (KinTek Corporation) Explorer software (61), which allowed us to test the ability of different reaction models to fit the data.

**Figure 6:**
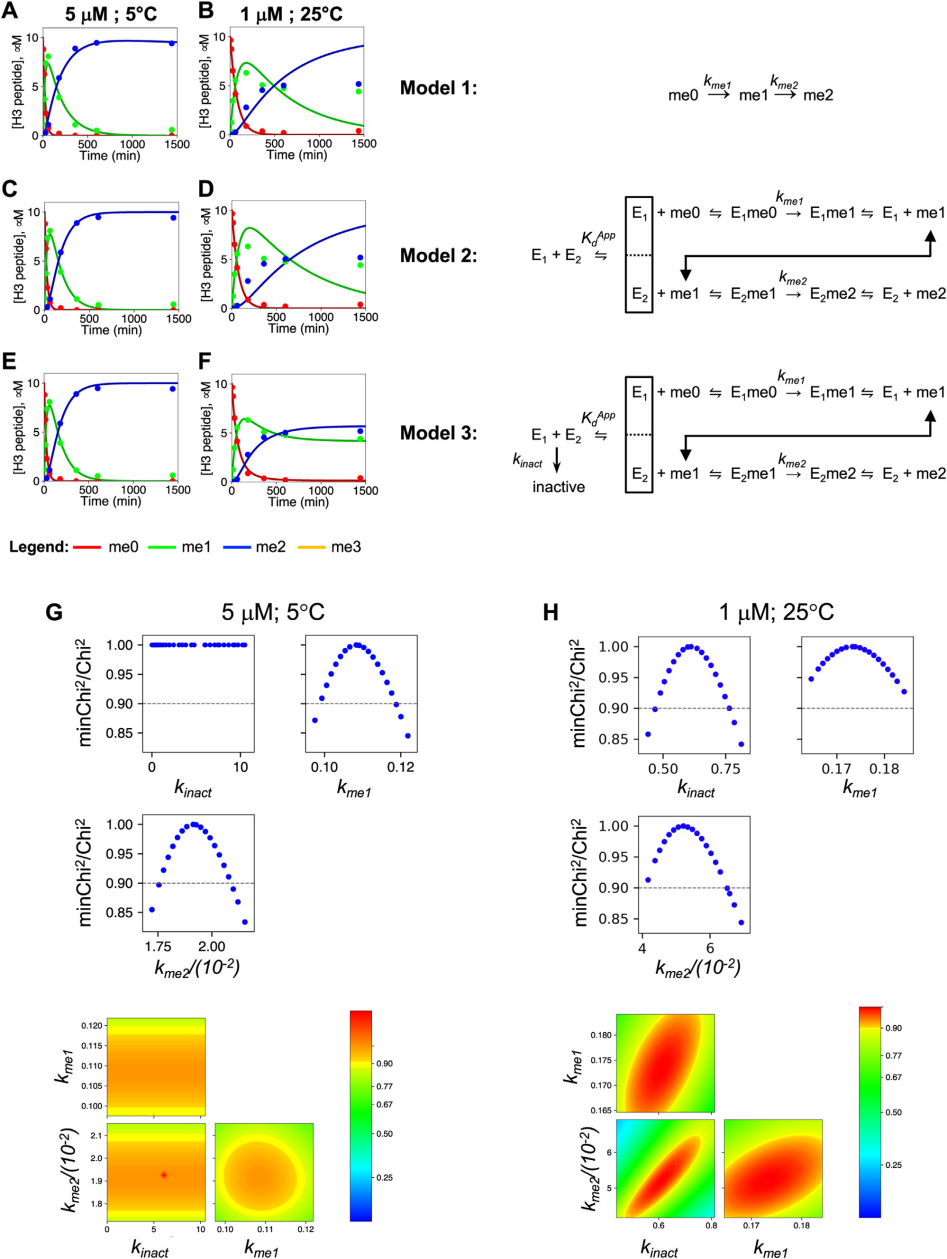
**Comparison of minimal enzymatic reaction pathways.***A*, *C*, and *E,* show the fits (*solid lines*) for the concentrations of each peptide species (me0, me1, or me2) for the same reaction time course, catalyzed by 5 μM MWRAD_2_ at 5 °C. Each panel shows the fits to the same data using model 1 (*A*), model 2 (*C*), or model 3 (*E*). *B*, *D*, and *F,* show fits for models 1 to 3, respectively, for the reaction time course catalyzed by 1 μM MWRAD_2_ at 25 °C. *G,* FitSpace confidence contour analysis for the reaction catalyzed by 5 μM MWRAD_2_ at 5 °C fit with model 3. *k*_*inact*_ is not constrained by the data, mainly because of the absence of detectable enzyme inactivation during the reaction time course at 5 °C. *H,* FitSpace confidence contour analysis of the fit of model 3 to the reaction catalyzed by 1 μM MWRAD_2_ at 25 °C. *k*_*inact*_ is now constrained by the data. MWRAD_2_, MLL core complex with WDR5, RbBP5, Ash2L, and two copies of DPY-30.

Using the simplest irreversible consecutive reactions model (Fig. 6, model 1), while acceptable fits were obtained for reaction progress curves collected at the highest concentration (5 μM) between temperatures 5 to 30 °C (5 °C is shown in Fig. 6*A*), the rest of the fits were poor (an example is shown in Fig. 6*B*). Since we previously showed that the complex uses a nonprocessive mechanism for multiple lysine methylation (36), we revised the model to incorporate binding of peptide substrate to the enzyme–AdoMet complex (E_1_) and release of the H3K4me1 product after the first methylation event, followed by binding of the H3K4me1 substrate to a distinct site on the enzyme (E_2_) for the dimethylation reaction. The latter step is predicated on our previous observation that the MLL1 core complex has a cryptic second active site independent of the SET domain that is required for the H3K4 dimethylation reaction (36, 62, 63). Since the binding and release rates of substrates and products are currently unknown, these values were fixed to be non–rate limiting. This model allowed us to incorporate an additional term to test the impact of reversible complex disassembly, which results in negligible activity of both enzymes under these assay conditions (Fig. 6, model 2) (36, 37). Initial values for the ratio (*k*_*off*_*/k*_*on*_) for complex assembly were set to be equal to the *K*_*d*_^*app*^ derived from each SV-AUC isotherm experiment.

**Figure 7:**
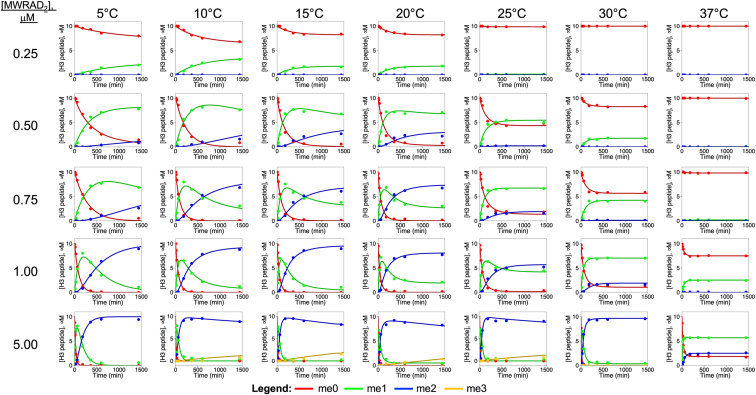
**Temperature and concentration dependence of MLL1 core complex enzymatic activity.** Time courses for reactions at the indicated MWRAD_2_ concentrations and temperatures were plotted and fit using model 3. Each time point represents the average from two independent experiments. Concentrations of each peptide species were plotted in *red* for H3K4me0, *green* for H3K4me1, and *blue* for H3K4me2. For reactions showing small amounts of H3K4me3 (*yellow*), model 3 was modified to incorporate an additional turnover step followed by product release. Note: Figure 6, *E* and *F* are reused in this figure, as they are the 5 μM MWRAD_2_ at 5 °C (*bottom left panel*) and 1 μM MWRAD_2_ at 25 °C (*fourth row*; *fifth column*) reaction time courses, respectively, fit with model 3. H3K4, histone H3 lysine 4; MLL, mixed lineage leukemia; MWRAD_2,_ MLL core complex with WDR5, RbBP5, Ash2L, and two copies of DPY-30.

The resulting simulations showed that adding a reversible complex disassembly step to the reaction scheme only modestly improved fits to the lower temperature data (Fig. 6*C*) but did not improve the fits of the higher temperature data (Fig. 6*D*). In addition, FitSpace confidence contour analysis (64) showed that the derived *k*_*off*_ value for the complex dissociation step was not constrained by the data (not shown), suggesting that the model is more complex. Closer examination of the high temperature data showed that several reactions failed to go to completion, suggesting the enzyme rapidly inactivates at higher temperatures (Fig. 7). We therefore revised the working model to incorporate an irreversible enzyme inactivation step (*k*_*inact*_) (Fig. 6, model 3). The resulting simulations resulted in good fits to both the low and high temperature datasets shown in Figure 6, *E* and *F*, respectively. In addition, FitSpace analysis showed that the derived pseudo–first-order rate constants for monomethylation (*k*_*me1*_) and dimethylation (*k*_*me2*_) reactions were reasonably well constrained by the data (Fig. 6, *G* and *H*). Furthermore, the rate of enzyme inactivation (*k*_*inact*_) was constrained by the data in the higher temperature experiments (Fig. 6*H*) but not in the lower temperature experiments (Fig. 6*G*), where enzyme inactivation is negligible. Figure 7 shows that the use of model 3 produces good fits for all datasets.

**Figure 8:**
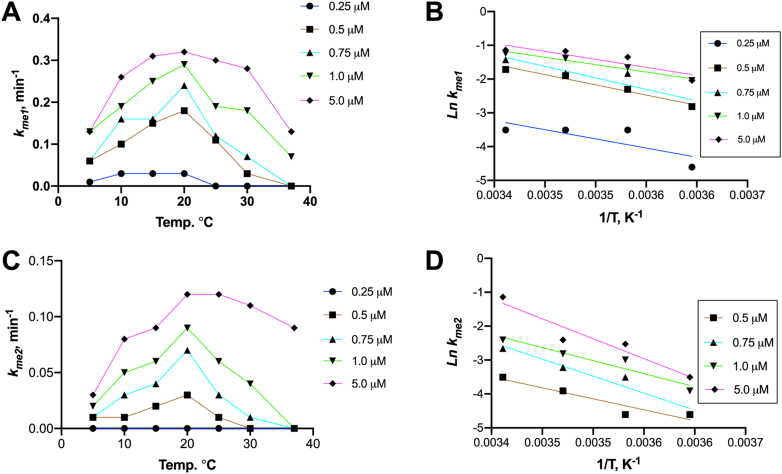
**Effect of temperature on MWRAD**_**2**_**enzymatic activity.***A* and *C,* rates of H3K4 monomethylation (*A*) and dimethylation (*C*) plotted as a function of temperature. Arrhenius behavior (defined as a doubling of the rate for every 10 °C increase in temperature) was observed between 5 and 20 °C for most concentrations. *B* and *D,* Arrhenius plots for H3K4 monomethylation (*B*) and dimethylation (*D*) for the data collected between 5 and 20 °C. The *lines* represent linear regression fits to the data collected at the indicated MWRAD_2_ concentrations. *E*_*a*_ values were obtained from the slope of the Arrhenius fits, where *slope = −*(*E*_*a*_*/R*) at each enzyme concentration. H3K4, histone H3 lysine 4; MWRAD_2,_ MLL core complex with WDR5, RbBP5, Ash2L, and two copies of DPY-30.

Based on these results, we then used the fits to model 3 to compare the impacts of temperature and concentration on the enzymatic activity of the MLL1 core complex (Fig. 8). The obtained pseudo–first-order rate constants for monomethylation (*k*_*me1*_), dimethylation (*k*_*me2*_), and the rate of enzyme inactivation (*k*_*inact*_) are summarized in Tables S3–S5, respectively. At most of the tested enzyme concentrations, activity increased linearly as the temperature increased from 5 to 20 °C (Fig. 8, *A* and *C*). However, above 20 °C, non-Arrhenius behavior was observed, as the rate of irreversible enzyme inactivation (*k*_*inact*_) rivaled or exceeded the rates of turnover (Tables S3–S5), resulting in reactions that failed to go to completion (Fig. 7). These results are consistent with the conclusions from the SV-AUC analysis, which suggested that as the complex dissociates at higher temperatures, one or more of the subunits undergoes an irreversible conformational change that is not competent for catalysis. We therefore plotted *k*_*me1*_ and *k*_*me2*_ rates (Ln(*k*_*n*)_) as a function of temperature (1/T) between 5 °C and 20 °C to fit the data to the Arrhenius equation (Fig. 8, *B* and *D*, respectively). Linear fitting of the Arrhenius plots revealed similar values for the energy of activation (*E*_*a*_) between the tested concentrations. The average *E*_*a*_ values were 10.9 ± 2.0 kcal K^−1^ mol^−1^ and 17.8 ± 4.7 kcal K^−1^ mol^−1^ for the monomethylation and dimethylation reactions, respectively.

The minimum enzyme concentration resulting in complete conversion into the monomethylated and then dimethylated forms was 1.0 μM at 15 °C (Fig. 7). Slightly higher activity was observed at the same enzyme concentration at 20 °C but with evidence of significant enzyme inactivation resulting in failure to go to completion. Increased concentration extended the range of temperatures under which complete conversion could be observed. For example, at 5 μM enzyme concentration, complete conversion of the peptide into the dimethylated form was observed between 5 °C and 30 °C, with evidence of modest H3K4 trimethylation activity (7–15%) between 10 °C and 25 °C (Fig. 7). However, at 37 °C, only ∼25% of the peptide was converted into the dimethylated form before the enzyme was completely inactivated.

## Discussion

In this investigation, we systematically characterized the hydrodynamic and kinetic properties of a reconstituted human MLL1 core complex under a variety of assay conditions. We found that complex assembly is highly concentration and temperature dependent. Consistent with the hypothesized hierarchical assembly pathway, we found that the holocomplex assembles through interactions between the MW and RAD_2_ subcomplexes and that this assembly is correlated with enzymatic activity. However, unexpectedly, we also found that the disassembled state of the complex is favored at physiological temperatures and at the submicromolar enzyme concentrations typically used in steady-state enzymatic assays (in which the substrate is in vast excess compared with the concentration of enzyme). We found that the complex disassembly results in rapid and irreversible enzyme inactivation under these conditions, likely because one or more individual subunits samples unproductive conformational states.

These results have immediate implications for the study of this and other multisubunit enzyme complexes as MWRAD_2_ activity assays and inhibition studies have been performed under a variety of temperatures and concentrations across laboratories. Based on our results, we recommend assays be conducted with at least 1 μM MLL1 core complex and at 15 °C when working *in vitro*. Concentrations below this threshold fail to go to completion, as do reactions above 15 °C (Fig. 7). Variation from these experimental conditions likely underlies variation in IC_50_ values for inhibitors targeting the WDR5 interaction motif—WDR5 interaction in different laboratories, making it difficult to identify the best inhibitors. For example, we previously found that an approximately fourfold change in [MWRAD_2_] resulted in an approximately eightfold change in the IC_50_ values obtained for inhibition of complex activity (65). It is therefore important to establish that the full complex is assembled and stable at the desired assay concentrations and temperatures over the duration of the experiment. It is most likely differences in MWRAD_2_ assembly state that underlies lab-to-lab variability in observed methylation rates for the SET1 family core complexes.

Verification of complex formation is also essential for obtaining reasonable *k*_*app*_ values for H3K4 methylation because SET1 family SET domains are slow monomethyltransferases in the absence of WDR5, RbBP5, and Ash2L (36). The presence of a disassembled species in activity assays could result in kinetic constants being underestimated, perhaps significantly, depending on the percentage of complex in this state. In addition, several SET1 family members (MLL1, MLL4, Setd1a, and Setd1b) gain the ability to multiply methylate H3K4 in the presence of WRAD_2_ (36, 66). Without prior knowledge of complex formation and stability at assay concentrations and temperature, it would be possible to lose multiple methylation of H3K4 for the SET1 complex under investigation. This effect, coupled with techniques that do not distinguish between lysine methylation states, such as ^3^H-mediated fluorography and fluorescence-coupled reactions could easily result in the omission of multiple methylation rates, or with them being accidentally grouped into the monomethylation rate estimate. This issue can be alleviated using previously methylated substrate peptides and by keeping observations within the linear range of a reaction but ideally, observing lysine methylation kinetics by lysine methyltransferases should be done with a method that distinguishes each methyl state, as well as quantifying the amounts of each species present in the reaction at each timepoint. Both quantitative and label-free MALDI-TOF MS (36, 66) and quantitative and continuous detection, ^13^C-methyl incorporation NMR (67) have proven to be valuable methods for lysine multiple methylation studies such as these.

**Figure 9:**
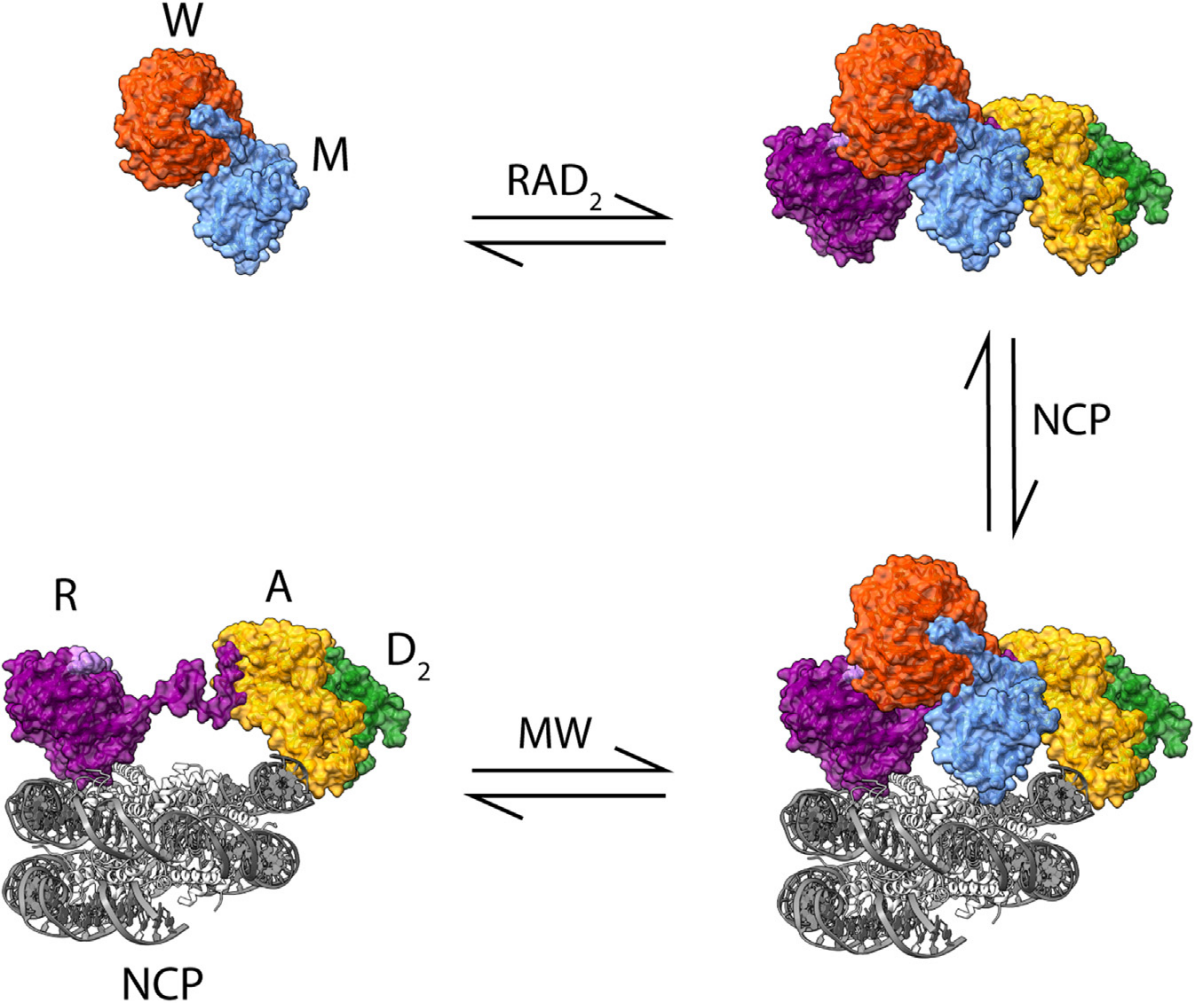
**Model of MWRAD**_**2**_**assembly on the nucleosome core particle (NCP).** MW can bind to RAD_2_ both in solution and prebound to NCP. The RAD_2_ subcomplex binds to nucleosome in the absence of MW, primarily through nonspecific DNA contacts. Figure made using Protein Data Bank ID: 7UD5 (79). MWRAD_2,_ MLL core complex with WDR5, RbBP5, Ash2L, and two copies of DPY-30.

While the concerns these results highlight for *in vitro* characterization of the MLL1 core complex activity can be addressed in the ways previously stated, they also lead directly to a question about its *in vivo* nature: how does this complex form at physiological temperature? Our studies of MWRAD_2_ at 37 °C show a strong tendency toward disassembly, one that could potentially be overcome by increasing the concentration of the complex components to many tens of micromolar, as suggested by the *S*_*W*_ isotherm results (Fig. 5*A*). This suggests a model in which the basal state of the complex is disassembled into MW and RAD_2_ subcomplexes. Given that RbBP5 and Ash2L can interact with nucleosomes on their own (data not shown), and their high concentration in cells compared with MLL1 (68), it seems likely that RAD_2_ interacts with nucleosomes first. Subsequently, MW, which exists at the C-terminal end of a long and flexible, intrinsically disordered region of the MLL1 primary sequence (Fig. S2) (69), swings in and binds to complete core complex assembly right on the nucleosome face when concentrations have reached the critical threshold (Fig. 9). This “swinging domain” mechanism would allow for very precise control of H3K4 methylation throughout the chromatin landscape and may explain why WRAD_2_ subunits exist in a vast stoichiometric excess relative to MLL1 (68). It is possible that additional factors are also required for a more stable complex formation, such as post-translational modifications of complex components (70). The ∼3700 missing residues of MLL1 in the construct studied here and/or the presence of additional subunits could also be responsible for conferring a more stable complex architecture in the cell. However, in support of a “mass action” mechanism, we have shown that the MLL1 core complex can be induced to undergo liquid–liquid phase separation *in vitro* (in preparation), where high local concentrations of complex subunits overcome the entropic barrier for complex assembly. Consistent with this mechanism, it has previously been demonstrated that MLL1 has a punctate distribution within the nucleus (71) and that it colocalizes with RNA polymerase II in transcription factories (72). Entry of the MLL1 core complex into dense regions such as this may allow sufficient activity against the chromatin substrate, providing a potential mechanism for spatial and temporal control of H3K4 methylation.

## Experimental procedures

### Protein expression and purification

Each of the human genes for the MLL1 SET domain (amino acids 3745–3969; UniProt ID: Q03164), WDR5 (amino acids 2–334; UniProt ID: P61964), RbBP5 (amino acids 1–538; UniProt ID: Q15291), and Ash2L (amino acids 1–534; UniProt ID: Q9UBL3-3) (73) were cloned into the pST44 polycistronic vector (74). The WDR5 subunit was cloned with an N-terminal 6x-Histidine tag followed by a tobacco etch virus protease cleavage site. Plasmids were transformed into Rosetta pLysS BL21 *Escherichia coli* cells and plated on LB agar supplemented with 50 μg/ml carbenicillin and 20 μg/ml chloramphenicol (both from Gold Biotechnology). Individual colonies were used to inoculate a seed culture of 50 ml of Terrific Broth II (MP Biomedicals), again supplemented with carbenicillin and chloramphenicol and grown overnight at 30 °C. About 20 ml of the seed culture was used to inoculate 1 l of Terrific Broth II media in baffled 2800 ml flasks, maintaining the antibiotic resistance. Cultures were then grown for 2 to 4 h at 37 °C and 200 RPM shaking until the absorbance reached ∼1 at 600 nm. Cultures were then chilled for 1 h at 4 °C followed by induction with 1 mM IPTG (Gold Biotechnology), after which cells were grown for an additional 20 to 22 h at 16 °C with constant shaking. Cells were harvested by centrifugation at 4 °C, and pellets were flash frozen in liquid nitrogen and stored at −80 °C until they could be lysed. Frozen cells were thawed and resuspended in 50 ml of lysis buffer (50 mM Tris–HCl, pH 7.5; 300 mM NaCl; 30 mM imidazole; 3 mM DTT, and 1 μM ZnCl_2_, supplemented with one tablet of EDTA-free protease inhibitor cocktail [Roche]), lysed with a microfluidizer, and cleared by centrifugation at 17,000 RPM at 4 °C for 30 min. The supernatant was diluted to 250 ml in buffer 1 (50 mM Tris–HCl, pH 7.5; 300 mM NaCl; 30 mM imidazole; 3 mM DTT, and 1 μM ZnCl_2_) and flowed over a HisTrap 5 ml nickel affinity column (GE) using an AKTA Purifier FPLC (GE) at a rate of 0.5 ml/min. Bound complex was washed with 10 column volumes of buffer 1 at 1 ml/min and then eluted with a 25-column volume linear gradient of buffer 2 (buffer 1 with 500 mM imidazole). Fractions containing the MWRA complex were pooled, supplemented with glutathione-*S*-transferase-6x-His-tobacco etch virus protease to a final concentration of 0.1 mg/ml and dialyzed against buffer 1 with three changes. The complex was then passed over a re-equilibrated HisTrap column, and fractions from the flow-through containing the cleaved MWRA sample were collected, concentrated by ultrafiltration using a 30 kDa cutoff membrane to ∼15 ml, and further purified by multiple rounds of SEC using a Superdex 200 (16/60) column (GE) pre-equilibrated with buffer 3 (20 mM Tris–HCl, pH 7.5; 300 mM NaCl; 1 mM Tris(2-carboxyethyl)phosphine; and 1 μM ZnCl_2_), with 5 ml sequential injections. The resulting fractions of pure MWRA were concentrated to <5 ml and a twofold molar excess of human DPY-30 (amino acids 1-99; UniProt ID: Q9C005), expressed and purified as previously described (36), was added to the sample. The resultant complex was purified with multiple rounds of SEC in buffer 3. Fractions containing purified MWRAD_2_ were concentrated to 12 mg/ml, aliquoted, flash frozen, and stored at −80 °C until use. Individual subunits for Bayesian experiments were purified as previously described (36).

### SV-AUC

#### Experimental set-up

All stock protein samples were thawed on ice, diluted to the desired concentration, and spun at 15,000 RPM for 15 min at 4 °C using a Thermo Scientific tabletop refrigerated centrifuge to remove any debris. Protein concentrations were measured with a NanoDrop spectrophotometer using the extinction coefficient *ε*^280^ of 248,954 M^−1^ cm^−1^, which was predicted from the amino acid sequence using ProtParam (Expasy.org) (75). About 100 or 400 μl of diluted protein samples were then loaded into AUC cells containing 3 mm or 12 mm two-sector charcoal-Epon centerpieces (Spin Analytical) assembled with quartz or sapphire windows. Matching buffer was loaded into the reference sector of each cell. AUC cells were then loaded into a Ti-60 4-hole Beckman-Coulter rotor that was pre-equilibrated to the specific run temperature for at least 4 h. Rotors were then inserted into the chamber of the centrifuge and allowed to re-equilibrate to experimental temperature for a minimum of 2 h before initiation of the run. SV-AUC was performed using a Beckman-Coulter Proteomelab XL-A analytical ultracentrifuge equipped with absorbance optics. Each run was preceded by a 3000 rpm wavelength scan to detect cell leakage and to select the appropriate wavelength to ensure a starting absorbance of between 0.25 and 1.2 absorbance units. Wavelengths at or near the maximal absorbance for aromatics of 280 nm or peptide backbone of 230 nm were selected, depending on the protein concentration and pathlength of the centerpiece. Without slowing the rotor, a method scan of 50,000 rpm was initiated, and 200 scans/cell were collected with the time interval between scans set to zero. Each experiment was replicated in duplicate or triplicate.

#### Data analysis

Lamm equation modeling of all SV-AUC results was performed using the continuous distribution (*c*(*s*)) method in SEDFIT (56). Maximum entropy (ME) regularization using a confidence level of *p* = 0.68 was performed to identify the most parsimonious distribution consistent with the data, and the fits for each experiment gave acceptable RMSD values ranging between 0.003 and 0.01. Density, viscosity, and partial specific volume values were estimated by inputting the temperature, buffer reagents, and amino acid sequences of all five complex components (assuming a DPY-30 dimer) into the SEDNTERP (https://bitc.sr.unh.edu/index.php/Main_Page) program (76), and the values used are listed in Table S6. The resulting *c*(*s*) distributions were displayed and further analyzed using GUSSI (https://www.utsouthwestern.edu/research/core-facilities/mbr/software/) (77). To determine the amount of holocomplex under each condition, distributions were integrated between *S* values 6.8 and 7.6, which represents one standard deviation from the mean *S* value of the holocomplex peak over all conditions, which was 7.2 ± 0.4. For binding analyses, *c*(*s*) distributions were integrated from 0.5 to 9.5 *S* to derive the corresponding signal-weighted average sedimentation coefficients (*s*_*w*_), which were plotted as a function of loading concentration at each temperature and fit with mass action law models using the program SEDPHAT (78).

For Bayesian analyses of *c*(*s*) distributions, expected sedimentation coefficients were derived from separate SV-AUC experiments of individual subunits or assembled subcomplexes, which were each run at concentrations ranging from 0.25 to 5 μM at 25 °C (the data for 0.25 μM runs are shown in Fig. S1). These values were then used in ME regularization as prior expectation restraints to give *c*^(*p*)^(*s*) distributions of the holocomplex at 25 °C. Prior expectations for subcomplexes or individual subunits were implemented as Gaussians in SEDFIT for Bayesian analysis, with a peak width of sigma = 0.2 *S* and centered at the weight-average *S* value of the main peak observed in the individual experiments with an amplitude of 0.05 absorbance units. Since the prior expected *S* values for WDR5 or RbBP5 overlapped when run in individual experiments, they were used as prior expectations in c^(*p*)^(*s*) distributions to test the concerted assembly mechanism with the same weight-average *S* value but with an amplitude that was doubled (Fig. 3*D*). Each *c*^(*p*)^(*s*) distribution was fit with the same prior expectation for MWRAD_2_, which used the weight-average *S* value determined at 25 °C and 0.25 μM with a width of sigma = 0.4 *S* and an amplitude of 0.3 absorbance units.

### Methyltransferase activity assay

MWRAD_2_ complex was assayed using a label-free quantitative MALDI-TOF mass spectrometry assay (36). Each 20 μl reaction consisted of varying concentrations of MWRAD_2_, 250 μM AdoMet, and reaction buffer (50 mM Tris, pH 9.0; 200 mM NaCl; 5% (v/v) glycerol; 1 μM ZnCl_2_; and 3 mM DTT), which were preincubated for 5 min at the experimental temperature in a thermocycler. Reactions were initiated by the addition of temperature-pre-equilibrated histone H3 peptide (residues 1–20, with an additional C-terminal GGK-biotin moiety) to a final concentration of 10 μM. At various time points, a 2 μl aliquot was removed and quenched by mixing with 2 μl of 1% TFA. Quenched reactions were stored at −20 °C until they could be analyzed. Samples were thawed, and 1 μl of each was mixed with 4 μl of α-cyano-4-hydroxycinnamic acid in 0.5% TFA and 50% acetonitrile. About 2 μl of this mixture for each time point was spotted onto a ground steel target plate and allowed to dry at room temperature for 3 to 12 h. Spectra were acquired on a Bruker Autoflex III MALDI-TOF mass spectrometer in reflectron mode. Each spectrum was the sum of at least 1000 individual laser shots, obtained from five different positions around the spot, with 200 shots at each position. Using FlexAnalysis software (Bruker), the intensities of the unmodified (*m/z* 2651 Da), monomethylated (*m/z* 2665 Da), dimethylated (*m/z* 2679 Da), and trimethylated (*m/z* 2693 Da) species were summed to obtain the total intensity. The relative amount of each species was then determined by dividing the intensity of each methylation state by the total intensity at each time point and multiplied by the starting substrate concentration (10 μM) to give the micromolar concentration of each methylation state. These data were then plotted as a function of time for kinetics analyses.

Fitting of the data was performed using the numerical integration of rate equations approach implemented in KinTek Explorer software, version 6.3 (61). For reaction models incorporating the complex dissociation step, the ratio (*k*_*off*_*/k*_*on*_) was constrained to be equal to estimated *K*_*d*_^*app*^ for complex dissociation at each temperature determined from the sedimentation velocity *s*_*w*_ isotherm analysis, with the *k*_*on*_ fixed at the limit of diffusion. All other nonvariable parameters were fixed with non–rate-limiting values. Confidence contour analysis using a *Chi*^*2*^ threshold of 0.9 was used to obtain estimates for the extent to which each variable parameter was constrained by the data.

### Statistical methods

Pearson’s linear correlation coefficient was used to assess relationships between H3K4 methylation rates and the biophysical parameters. The significance of the Pearson correlation coefficient was evaluated using a *t* test in XLStat (Addinsoft) software.

## Data availability

The raw SV-AUC data and MALDI-TOF time-course methyltransferase data used for analysis are available upon reasonable request.

## Supporting information

This article contains supporting information (69).

## Conflict of interest

M. S. C. owns stock and serves on the Consultant Advisory Board for Kathera Bioscience, Inc, the makers of antifungal technologies. M. S. C. also holds US patents (8,133,690), (8,715,678), and (10,392,423) for compounds and methods for inhibiting SET1–MLL family complexes.
