# Supplementary material for: Hierarchical assembly of the MLL1 core complex regulates H3K4 methylation and is dependent on temperature and component concentration
Source: J Biol Chem. 2023 Jan 6;299(2):102874. doi: 10.1016/j.jbc.2023.102874 (PMC9939731; doi:10.1016/j.jbc.2023.102874)
Supplement: Supporting information [file mmc1.docx]

**Hierarchical assembly of the MLL1 core complex regulates H3K4 methylation and is dependent on temperature and component concentration**

Kevin E. W. Namitz^1, 2^, Song Tan^3^, and Michael S. Cosgrove^1^*

Supporting information

^1^State University of New York (SUNY) Upstate Medical University, Department of Biochemistry and Molecular Biology, Syracuse, New York.

^2^ Current Address: Penn State University, Department of Chemistry, University Park, Pennsylvania

^3^ Penn State University, Department of Biochemistry and Molecular Biology, University Park, Pennsylvania

*To whom correspondence should be addressed: Michael S. Cosgrove, Ph.D., Department of Biochemistry and Molecular Biology, SUNY Upstate Medical University, 750 East Adams Street, Syracuse, NY 13210, Phone: (315) 464-7751, E-mail: [cosgrovm@upstate.edu](mailto:cosgrovm@upstate.edu)


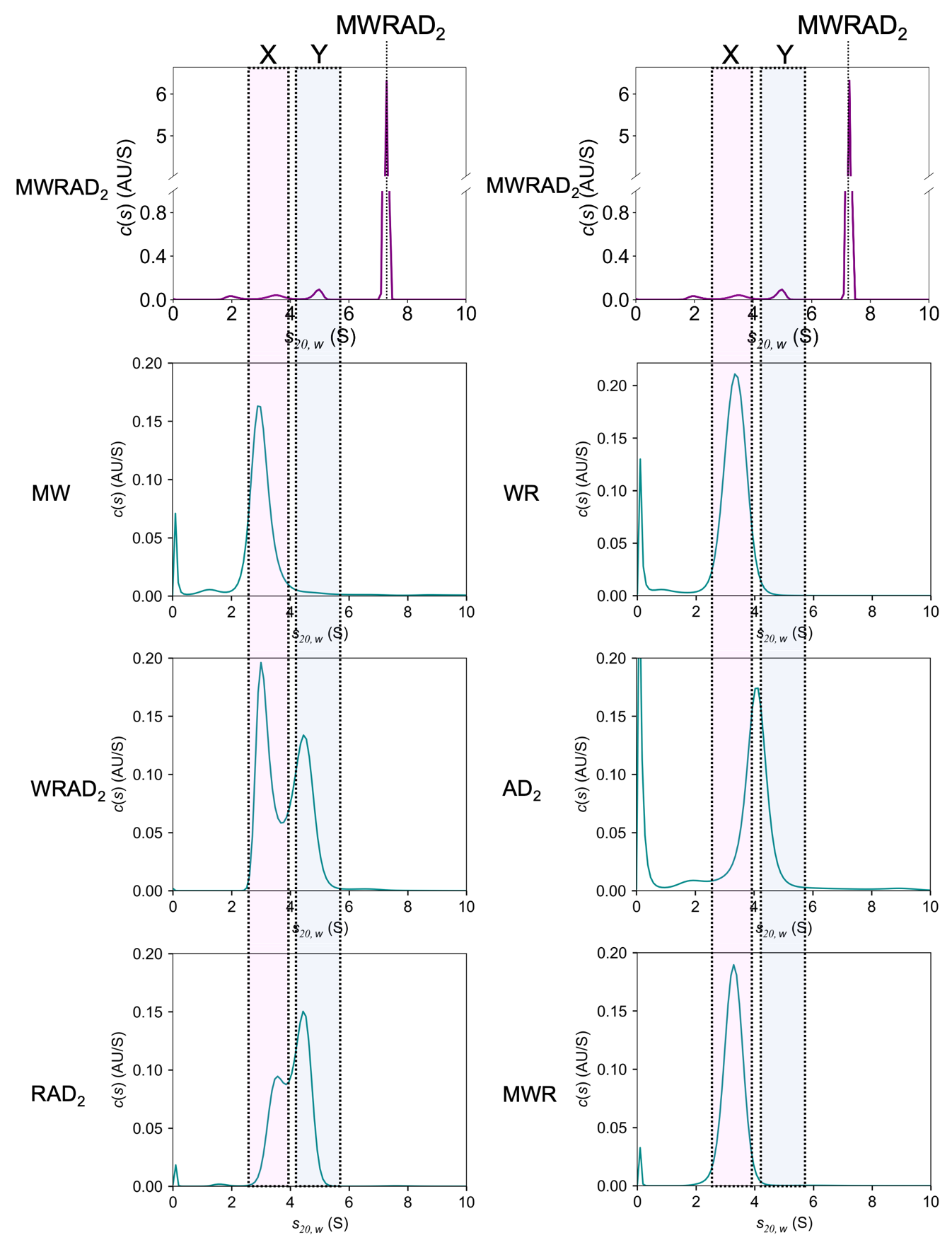


**Figure S1 – Namitz, Tan and Cosgrove**

**Figure S2 – Namitz, Tan and Cosgrove**

**Table S1:** Summary of *S*-values for MLL1 core complex subunits and subcomplexes from SV-AUC experiments at 25° C.

| **Protein** | ***s_20,w_***  **(5 µM)** | ***s_20,w_***  **(1 µM)** | ***s_20,w_***  **(0.25 µM)** |
| --- | --- | --- | --- |
| **MWRAD_2_** | 7.2 | 7.2 | 6.9 |
| **WRAD_2_** | 5.5 | 5.2 | 4.4 |
| **RAD_2_** | 5.2 | 5.2 | 4.4 |
| **MWR** | 4.8 | 3.6 | 3.3 |
| **WR** | 4.1 | 3.3 | 3.2 |
| **AD_2_** | 4.1 | 4.1 | 4.1 |
| **MW** | 3.9 | 3.8 | 3.0 |
| **M** | 2.3 | 2.3 | 2.3 |
| **W** | 3.2 | 3.2 | 3.2 |
| **R** | 3.4 | 3.4 | 3.4 |
| **A** | 3.7 | 3.7 | 3.7 |
| **D_2_** | 1.9 | 1.9 | 1.9 |

**Table S2:** Summary of the amount of holo-MLL1 core complex from SV-AUC experiments at the indicated concentrations and temperatures^a^**.**

| **[MWRAD_2_]**  **(µM)** | **5°C** | **10°C** | **15°C** | **20°C** | **25°C** | **30°C** | **37°C** |
| --- | --- | --- | --- | --- | --- | --- | --- |
| **0.25** | 83 (3.0) | 79 (2.0) | 63 (9.0) | 56 (3.0) | 48 (1.0) | 8 (0.7) | 3.0 (2.0) |
| **0.5** | 86 (0.6) | 88 (3.0) | 85 (1.0) | 81 (2.0) | 68 (5.0) | 43 (2.0) | 1.0 (0.3) |
| **0.75** | 91 (0.7) | 90 (0.4) | 89 (0.4) | 86 (3.0) | 78 (0.0) | 55 (1.0) | 0.9 (0.1) |
| **1.0** | 90 (0.5) | 83 (3.0) | 82 (2.0) | 81 (1.0) | 76 (0.5) | 56 (5.0) | 1.3 (0.5) |
| **5.0** | 88 (0.3) | 92 (0.4) | 81 (4.0) | 88 (2.0) | 91 (0.4) | 57 (6.0) | 2.1 (2.0) |

^a^ Each value represents the mean percent (±S.D.) of holo-MLL1 core complex signal sedimenting between 6.8-7.6 *S* out of the total integrated signal of all species at each of the indicated loading concentrations and temperatures. Each experimental condition was conducted in duplicate or triplicate.

**Table S3.** Pseudo-first order rate constants for H3K4 monomethylation (*k_me1_*) catalyzed by MWRAD_2_ at the indicated concentration and temperature^*^

| Temperature: | 5°C | 10°C | 15°C | 20°C | 25°C | 30°C | 37°C |
| --- | --- | --- | --- | --- | --- | --- | --- |
| [MWRAD_2_], µM | *k_me1,_ min^-1^* | *k_me1,_ min^-1^* | *k_me1,_ min^-1^* | *k_me1,_ min^-1^* | *k_me1,_ min^-1^* | *k_me1,_ min^-1^* | *k_me1,_ min^-1^* |
| 0.25 | 0.01 ± 0.01 | 0.03 ± 0.02 | 0.03 ± 0.04 | 0.03 ± 0.03 | 1. ± 0.05 | N/A^a^ | N/A |
| 0.5 | 0.06 ± 0.01 | 0.10 ± 0.02 | 0.15 ± 0.03 | 0.18 ± 0.03 | 0.11 ± 0.05 | 0.03 ± 0.02 | N/A |
| 0.75 | 0.06 ± 0.01 | 0.16 ± 0.02 | 0.16 ± 0.02 | 0.24 ± 0.02 | 0.12 ± 0.04 | 0.07 ± 0.05 | 0.00 ± 0.02 |
| 1.0 | 0.13 ± 0.01 | 0.19 ± 0.04 | 0.25 ± 0.04 | 0.29 ± 0.04 | 0.19 ± 0.04 | 0.18 ± 0.04 | 0.07 ± N.D.^b^ |
| 5.0 | 0.13 ± 0.02 | 0.26 ± 0.04 | 0.31 ± 0.04 | 0.32 ± 0.04 | 0.30 ± 0.04 | 0.28 ± 0.04 | 0.13 ± 0.06 |

* Each is the rate constant +/- the standard error estimate (95% confidence interval) derived from nonlinear regression fitting of the data to *Model 3*. Duplicate measurements were made for each time point.

^a^ N/A, Not applicable – no methylation observed under the indicated condition.

^b^ N.D., standard error estimates are not defined.

**Table S4.** Pseudo-first order rate constants for H3K4 dimethylation (*k_me2_*) catalyzed by MWRAD_2_ at the indicated concentration and temperature^*^

| Temperature: | 5°C | 10°C | 15°C | 20°C | 25°C | 30°C | 37°C |
| --- | --- | --- | --- | --- | --- | --- | --- |
| [MWRAD_2_], µM | *k_me2,_ min^-1^* | *k_me2,_ min^-1^* | *k_me2,_ min^-1^* | *k_me2,_ min^-1^* | *k_me2,_ min^-1^* | *k_me2,_ min^-1^* | *k_me2,_ min^-1^* |
| 0.25 | N/A^a^ | N/A | N/A | N/A | N/A | N/A | N/A |
| 0.5 | 0.01 ± 0.00 | 0.01 ± 0.01 | 0.02 ± 0.01 | 0.03 ± 0.01 | 0.01 ± 0.04 | N/A | N/A |
| 0.75 | 0.01 ± 0.00 | 0.03 ± 0.01 | 0.04 ± 0.01 | 0.07 ± 0.02 | 0.03 ± 0.02 | 0.01 ± 0.06 | N/A |
| 1.0 | 0.02 ± 0.00 | 0.05 ± 0.02 | 0.06 ± 0.02 | 0.09 ± 0.03 | 0.06 ± 0.04 | 0.04 ± 0.01 | N/A |
| 5.0 | 0.03 ± 0.01 | 0.08 ± 0.01 | 0.09 ± 0.01 | 0.12 ± 0.02 | 0.12 ± 0.02 | 0.11 ± 0.02 | 0.09 ± 0.06 |

* Each is the rate constant +/- the standard error estimate (95% confidence interval) derived from nonlinear regression fitting of the data to *Model 3*. Duplicate measurements were made for each time point.

^a^ N/A, Not applicable – no methylation observed under the indicated condition.

**Table S5.** Summary of the MWRAD_2_ irreversible enzyme inactivation (*k_inact_*) rates.

| Temperature: | 5°C | 10°C | 15°C | 20°C | 25°C | 30°C | 37°C |
| --- | --- | --- | --- | --- | --- | --- | --- |
| [MWRAD_2_], µM | *k_inact,_ min^-1^* | *k_inact,_ min^-1^* | *k_inact,_ min^-1^* | *k_inact,_ min^-1^* | *k_inact,_ min^-1^* | *k_inact,_ min^-1^* | *k_inact,_ min^-1^* |
| 0.25 | 0.01 ± 0.02 | 0.02 ± 0.02 | 0.04 ± 0.08 | 0.05 ± 0.05 | 0.08 ± 1.07 | >1700^b^ | >2000^b^ |
| 0.5 | 0.01 ± 0.01 | 0.002 ± 0.01 | 0.01 ± 0.02 | 0.02 ± 0.01 | 0.06 ± 0.04 | 0.09 ± 0.07 | >390^b^ |
| 0.75 | 0.00 ± 0.01 | 0.009 ± 0.01 | 0.02 ± 0.01 | 0.03 ± 0.01 | 0.04 ± 0.03 | 0.09 ± 0.08 | 0.13 ± 1.00 |
| 1.0 | 0.00 ± N.D.^a^ | 0.01 ± 0.02 | 0.01 ± 0.01 | 0.04 ± 0.02 | 0.04 ± 0.03 | 0.07 ± 0.03 | 0.24 ± 0.11 |
| 5.0 | 0.01 ± 0.01 | 0.05 ± 0.02 | 0.00 ± 0.00 | 0.09 ± 0.02 | 0.07 ± 0.03 | 0.07 ± 0.02 | 0.21 ± 0.13 |

* Each is the rate constant +/- the standard error estimate (95% confidence interval) derived from nonlinear regression fitting of the data to *Model 3*. Duplicate measurements were made for each time point.

^a^ N.D., error estimates are not defined.

^b^ *k*_inact_ lower bound. In Kintek Explorer software, *k_me1_* was fixed to the value predicted by the Arrhenius equation at the indicated temperature and *k*_inact_ was floated to estimate the lower bound required for the observed loss of activity.

**Table S6.** Summary of density, viscosity, and partial specific volume estimates for the MLL1 core complex^a^

| **Temperature**  **(°C)** | **Density** | **Viscosity** | **Partial Specific Volume (v_bar_)** |
| --- | --- | --- | --- |
| **5** | 1.0129 | 0.01569 | 0.724 |
| **10** | 1.0126 | 0.01351 | 0.726 |
| **15** | 1.0120 | 0.01176 | 0.728 |
| **20** | 1.0111 | 0.01035 | 0.730 |
| **25** | 1.0099 | 0.00920 | 0.733 |
| **30** | 1.0085 | 0.00824 | 0.735 |
| **37** | 1.0062 | 0.00714 | 0.738 |

^a^ Density and viscosity values were derived from inputting buffer components into SEDNTERP (76) at the indicated temperatures. v_bar_ at each temperature was calculated from the MWRAD_2_ amino acid sequence using SEDNTERP (76).

**Supporting information Figure Legends**

**Figure S1: Determination of expected subcomplex S values for Bayesian analyses.** SV-AUC analysis of 5 μM MLL1 core complex was performed at 25°C with the resulting c(s) plot shown in the top two panels. The position of the holo-complex (MWRAD_2_) at 7.2 S is indicated. The range of positions for the unidentified subcomplex peaks X and Y S are shown in the dotted rectangles. Since each subcomplex peak represented ~ 5% of the total absorbance, we estimated these subcomplexes to be present in the sample at a concentration of ~0.25 μM. We then mixed stoichiometric amounts of individual subunits for each of the indicated subcomplexes at a final concentration of 0.25 μM and performed SV-AUC at 25°C. The resulting c(s) plots for each of the indicated subcomplexes are shown. While unambiguous assignment of the subcomplex peaks could not be determined from direct comparison of c(s) plots, the peak positions can serve as powerful restraints in Bayesian analyses of putative assembly schemes. The position of the largest peak in each subcomplex run was used as a prior expectation in the Bayesian analyses reported in Fig. 3. The peak positions of each subcomplex or subunit at 5, 1 and 0.25 μM are summarized in Table S1.

**Figure S2: The catalytic module of the MLL1 core complex exists at the end of a long-flexible intrinsically disordered region in the primary sequence of MLL1.** IUPred disorder prediction (79) for full-length MLL1 (Uniprot # Q03164). Uniprot sub-domain boundaries are shown in the schematic above.
